# Closed Genome Sequences of Clinical Neisseria gonorrhoeae Strains Obtained from Combined Oxford Nanopore and Illumina Sequencing

**DOI:** 10.1128/MRA.00072-19

**Published:** 2019-02-28

**Authors:** Richard C. White, Manolito Torralba, Kelly Colt, Frank Harrison, Karrie Goglin, Kevin Nguyen, Roshan D’Souza, Claire C. Bristow, Olivia Ellis, Olusegun O. Soge, Jeffrey D. Klausner, Derrick E. Fouts

**Affiliations:** a J. Craig Venter Institute, Rockville, Maryland, USA; b J. Craig Venter Institute, La Jolla, California, USA; c Division of Infectious Diseases and Global Public Health, Department of Medicine, University of California, San Diego, California, USA; d Department of Environmental Health Sciences, Fielding School of Public Health, University of California, Los Angeles, California, USA; e Department of Global Health, University of Washington, Seattle, Washington, USA; f Department of Medicine, University of Washington, Seattle, Washington, USA; g Division of Infectious Diseases, Department of Medicine, University of California, Los Angeles, California, USA; Queens College

## Abstract

Neisseria gonorrhoeae is the etiological agent of gonorrhea, the second most common notifiable disease in the United States. Here, we used a hybrid approach combining Oxford Nanopore Technologies MinION and Illumina MiSeq sequencing data to obtain closed genome sequences of nine clinical N. gonorrhoeae isolates.

## ANNOUNCEMENT

Neisseria gonorrhoeae is the causative agent of gonorrhea, the second most commonly reported notifiable disease in the United States (1). Gonorrhea poses a major public health threat, with emerging resistance to nearly all available antibiotics, including first-line dual therapy with azithromycin and ceftriaxone. Whole-genome sequencing (WGS) has become increasingly useful for tracking the spread and elucidating mechanisms of antimicrobial resistance (2–5). Prior to submission of our closed genomes to GenBank, there were 18 complete genome assemblies in the NCBI Reference Sequence Database (6). We report 9 new complete genomes spanning multiple sequence types.

N. gonorrhoeae isolates collected from symptomatic individuals for routine surveillance in Los Angeles, USA, were chosen for WGS. N. gonorrhoeae isolates were streaked onto Thayer-Martin chocolate agar plates (Hardy Diagnostics, Santa Maria, CA) and grown overnight at 37°C in a humidified 5% CO_2_ environment. For MinION sequencing, high-molecular-weight genomic DNA was prepared from bacteria scraped from Thayer-Martin agar using the Gentra Puregene Yeast/Bact. kit (Qiagen catalog number 158567) using manufacturer specifications. The Illumina sequencing libraries were prepared using the TruSeq library kit with bead-based size selection and were loaded onto an Illumina MiSeq flow cell. Sequencing was performed with a MiSeq reagent kit v3 in a 2 × 300-bp paired-end format. The MinION sequencing libraries were prepared using the rapid barcoding kit (catalog number SQK-RBK004) and loaded onto a single MinION R9.4 flow cell. Oxford Nanopore Technologies (ONT) reads were base-called with Albacore v2.0.2, generating 2.4 Gbp of data. Raw and trimmed Illumina reads were assessed using FastQC v0.11.7, and quality trimming was performed using Trimmomatic v0.32 (7) with the following settings: ILLUMINACLIP, TruSeq3-PE-2.fa:2:30:10; LEADING, 3; TRAILING, 3; SLIDINGWINDOW, 4:24; and MINLEN, 60. ONT reads were demultiplexed and quality-trimmed using Porechop v0.2.3 (8) with default settings. A hybrid Illumina-ONT *de novo* assembly was performed using the Unicycler v0.4.7 pipeline (9) in normal mode. Pilon v1.22 (10) was used iteratively (first using the flag --fix bases, then --fix all) to polish the assemblies with Illumina reads until no additional changes could be made. Detailed assembly statistics are provided in Table 1.

**TABLE 1:** Complete genome assembly statistics for the N. gonorrhoeae strains

	Data for strain:								
Feature	FQ01	FQ02	FQ04	FQ20	FQ35	FQ36	FQ48	FQ82	FQ84
BioSample ID^*a*^	SAMN10395998	SAMN10395999	SAMN10396000	SAMN10396003	SAMN10395917	SAMN10395916	SAMN10395915	SAMN10395914	SAMN10395918
Illumina read SRA accession no.	SRR8457072	SRR8457069	SRR8457068	SRR8457071	SRR8457070	SRR8457065	SRR8457064	SRR8457067	SRR8457066
ONT read SRA accession no.	SRR8457081	SRR8457080	SRR8457079	SRR8457078	SRR8457077	SRR8457076	SRR8457075	SRR8457074	SRR8457073
Illumina read count (millions) (coverage [×])	2.31 (408)	2.81 (509)	2.39 (432)	2.06 (372)	1.61 (291)	1.97 (356)	2.15 (390)	2.59 (455)	0.41 (74)
ONT read count (thousands) (coverage [×])	40.9 (66)	9.8 (19)	6.7 (38)	67.3 (134)	101.3 (206)	65.2 (116)	29.9 (66)	140.9 (293)	21.6 (51)
No. of circular contigs	3	2	2	2	2	2	2	2	2
Chromosome length (bp)	2,218,440	2,228,346	2,217,528	2,232,677	2,232,230	2,218,771	2,229,997	2,217,835	2,217,429
Plasmid length (bp)	4,207; 39,053	4,207	4,207	4,207	4,207	4,228	4,153	4,207	4,207
GenBank accession no. (chromosome)	CP034032	CP034030	CP034028	CP034026	CP034024	CP034022	CP034020	CP034018	CP034016
GenBank accession no. (plasmids)	CP034033, CP034034	CP034031	CP034029	CP034027	CP034025	CP034023	CP034021	CP034019	CP034017
GC content (%)	52.39	52.35	52.45	52.38	52.38	52.42	52.35	52.44	52.45
MLST	8122	9363	7363	13149	13149	1901	1901	7363	7363
NG-MAST	292	2992	7574	3506	3506	7631	8476	7574	7574
NG-STAR	299	63	1420 (novel)	1419 (novel)	1419 (novel)	560	756	540	540

a ID, identification number.

The genomes were annotated using the NCBI Prokaryotic Genome Annotation Pipeline (PGAP) (6, 11). Multilocus sequence typing (MLST) and N. gonorrhoeae sequence typing for antimicrobial resistance (NG-STAR) were determined *in silico* using LOCUST (12) with default settings and are provided in Table 1. N. gonorrhoeae multiantigen sequence typing (NG-MAST) was determined *in silico* using NGMASTER (13) with default settings and is provided in Table 1. Novel NG-STAR profiles were confirmed via PCR (14, 15) and Sanger sequencing (Genewiz, South Plainfield, NJ) and uploaded to the NG-STAR database (16).

This study highlights the value of long-read sequencing in both detecting and closing plasmid sequences, as circular plasmids have been detected in only 22% of the 18 closed N. gonorrhoeae genomes, yet the cryptic plasmid is present in over 96% of isolates (17). We detected and completely assembled the N. gonorrhoeae cryptic plasmid in all nine isolates and detected and assembled the conjugative plasmid in strain FQ01 (Table 1).

### Data availability.

The complete genome sequences and the raw sequencing reads have been deposited in GenBank and the NCBI Sequence Read Archive (SRA), respectively, and are available under the accession numbers listed in Table 1.
